# Mad Dog

**Published:** 1868-09

**Authors:** 


### MAD DOG.

Scarce a day passes which does not chronicle some new or old cure for Hydrophobia with the most amazing confidence when it is really doubtful whether a dozen persons, or six, or one, has ever recovered from an actual attack since the world began. To be bitten by a mad dog, let alone one only supposed to be mad and to have an actual attack of hydrophobia, are things as different as possible, for twenty persons may be bitten by an animal actually rabid, and but one out of the twenty will have an attack. Recently a man and wife were bitten by a dog in Jersey City ; the husband died in horrible convulsions in a few days, while the wife is in good health. Cases are recorded seeming to show that imagination or alarm may induce convulsions and death apparently hydrophobic. Two things ought to be promptly done in all cases : do something, do anything on the instant as a means of quieting the patient's mind, and have the family physician called in as few seconds as possible. Presence of mind and calmness on the part of attendants is of invaluable service. Every reader should carry with him this one practical idea during his life—As far as chemical analysis has progressed up to this date, all bites and stings of a poisonous kind affect the system by the acid nature of the venom, which enters the blood instantaneously ; every one may know that an alkali antagonizes an acid instantly ; the strongest familiar alkali is hartshorn, or in another form, "smelling salts ;" then wet a rag in the spirits, apply it to the wound and keep it wet until the physician arrives, and then turn over the responsibility to him. But suppose you have no hartshorn, then make a poultice of fresh wood ashes and water, because this also is an alkali. If a lady is bitten on her way to church use the smelling salts as a poultice by dissolving it in spirits; or if dry, and there are no spirits at hand, nor even a drop of water, make it into powder with a stone or any thing else, dampen it with the saliva, lay it on the wound, then cover it with a pocket-handkerchief, until the physician arrives; these proceedings will not only afford a measure of satisfaction to the patient, soothing and imparting confidence, but in reality they are the most efficient means known to science for antagonizing poisonous effects ; but not to burden the memory too much, it may be added that the hartshorn not only antagonizes the poison by its nullifying powers, but it "smarts" whenever applied to a broken skin, this smarting induces irritation, that is, it reddens the skin all around, which means that it draws the blood to the surface, keeping it from entering the general circulation. It may then occur to the reflecting reader, that if around the bite or sting, for an inch or two, the skin were scratched to bleeding with the point of a knife or even the finger nails, there would be an increased amount of irritation and a proportionately greater safety insured.

